# Effect of maternal gestational weight gain on offspring DNA methylation: a follow-up to the ALSPAC cohort study

**DOI:** 10.1186/s13104-015-1286-6

**Published:** 2015-07-29

**Authors:** Jon Bohlin, Bettina K Andreassen, Bonnie R Joubert, Maria C Magnus, Michael C Wu, Christine L Parr, Siri E Håberg, Per Magnus, Sarah E Reese, Camilla Stoltenberg, Stephanie J London, Wenche Nystad

**Affiliations:** Division of Epidemiology, Norwegian Institute of Public Health, Marcus Thranes gate 6, P.O. Box 4404, 0403 Oslo, Norway; Department of Molecular Biology, Institute of Clinical Medicine, University of Oslo, Oslo, Norway; National Institute of Environmental Health Sciences, MD A3-05, PO Box 12233, Research Triangle Park, NC 27709 USA; Public Health Sciences Division, Fred Hutchinson Cancer Research Center, Seattle, WA 98109 USA

## Abstract

**Background:**

Several epidemiologic studies indicate that maternal gestational weight gain (GWG) influences health outcomes in offspring. Any underlying mechanisms have, however, not been established. A recent study of 88 children based on the Avon Longitudinal Study of Parents and Children (ALSPAC) cohort examined the methylation levels at 1,505 Cytosine-Guanine methylation (CpG) loci and found several to be significantly associated with maternal weight gain between weeks 0 and 18 of gestation. Since these results could not be replicated we wanted to examine associations between 0 and 18 week GWG and genome-wide methylation levels using the Infinium HumanMethylation450 BeadChip (450K) platform on a larger sample size, i.e. 729 newborns sampled from the Norwegian Mother and Child Cohort Study (MoBa).

**Results:**

We found no CpG loci associated with 0–18 week GWG after adjusting for the set of covariates used in the ALSPAC study (i.e. child’s sex and maternal age) and for multiple testing (*q* > 0.9, *both* 1,505 *and* 473,731 *tests*). Hence, none of the CpG loci linked with the genes found significantly associated with 0–18 week GWG in the ALSPAC study were significant in our study.

**Conclusions:**

The inconsistency in the results with the ALSPAC study with regards to the 0–18 week GWG model may arise for several reasons: sampling from different populations, dissimilar methylome coverage, sample size and/or false positive findings.

**Electronic supplementary material:**

The online version of this article (doi:10.1186/s13104-015-1286-6) contains supplementary material, which is available to authorized users.

## Background

Recent genome-wide DNA methylation mapping technologies have resulted in an increasing number of studies examining epigenetic effects on offspring from various maternal exposures during pregnancy. For instance, results from the Norwegian Mother and Child Cohort Study (MoBa) indicate that maternal smoking during pregnancy influences methylation patterns in the offspring [1]. Furthermore, findings from MoBa show an association between patterns of methylation in the offspring and birth weight [2]. Although maternal gestational weight gain (GWG) has been associated with offspring’s health and development during childhood [3, 4] limited knowledge is currently available regarding epigenetic effects on offspring from maternal GWG.

Morales et al. conducted an epigenetic inquiry of putative effects from maternal pre-pregnancy BMI and GWG in cord blood DNA of 88 newborns by using data from the Avon Longitudinal Study of Parents and Children (ALSPAC) cohort [5]. They examined 807 candidate genes (1,505 CpG probes) for cancer using the Illumina GoldenGate Genotyping Assay. Several time intervals for GWG in pregnancy were tested, and a significant association between weeks 0–18 GWG, and a set of CpG probes linked to the genes: *MMP7*, *KCNK4*, *TRPM5* and *NFKB1* were reported. Morales et al. did not succeed in replicating their results in 170 non-overlapping ALSPAC subjects and encouraged more research based on larger studies and genome-wide DNA methylation data [5]. Hence, the aim of the present work was to explore the findings from Morales et al. using genome-wide DNA methylation data as well as determine the presence of any novel associations between newborn methylome CpG loci and maternal 0–18 week GWG. DNA methylation was measured in cord blood using the Infinium HumanMethylation450 BeadChip (450K), from 729 newborns participating in the MoBa cohort.

## Findings

### GWG models as described in the ALSPAC study

Morales et al. found a statistically significant association with probes linked to the genes *MMP7*, *KCNK4*, *TRPM5* and *NFKB1* for the model describing 0–18 week GWG. They tested the following covariates: child’s sex, ethnic background, mode of delivery, maternal age, parity, maternal smoking in pregnancy and occupation, but found only maternal age and offspring sex to be of importance with respect to the results. We ran a similar genome-wide model for the 473,731 probes on the Infinium HumanMethylation450 BeadChip (sex chromosomes excluded) that overlapped with the genes represented on the Illumina GoldenGate Genotyping Assay examined by Morales et al. [5]. We adjusted for child’s sex and maternal age in accordance with the model presented by Morales et al. A full model containing the covariates child’s sex, mode of delivery, maternal age, parity, maternal education and maternal daily smoking during pregnancy (ethnic background was irrelevant due to a highly homogeneous population) was also fitted but since there were no differences with respect to significant findings (see Additional file 1; Table 1) we present the simpler model, in accordance with the study by Morales et al. Hence, we regressed CpG loci against 0–18 week GWG adjusted for child’s sex and maternal age. The genomic inflation factor (GIF) λ [6] was used as an indicator of hidden confounders and for model assessment [7, 8].

**Table 1:** Summary statistics for the covariates used in the 0–18 weeks GWG regression models, n = 729

Covariate	Estimate
Child’s sex, male	48.8%
Mean age of mother at birth (years)	30.1 (95% CI 29.8–30.4)
Mean 0–18 weeks GWG (kg/week)	0.16 (95% CI 0.14–0.17)
Not completed high-school	7.1%
Completed high school	33.6%
College/university	41.8%
College/university more than 4 years	17.1%
Maternal daily smoking, yes (8 missing)	12.5%
Caesarian section, yes	6%
1st	40.7%
2nd	39.5%
3rd	14.1%
4th or more	5.6%

### Our results compared to those of the ALSPAC study

**Fig. 1:**
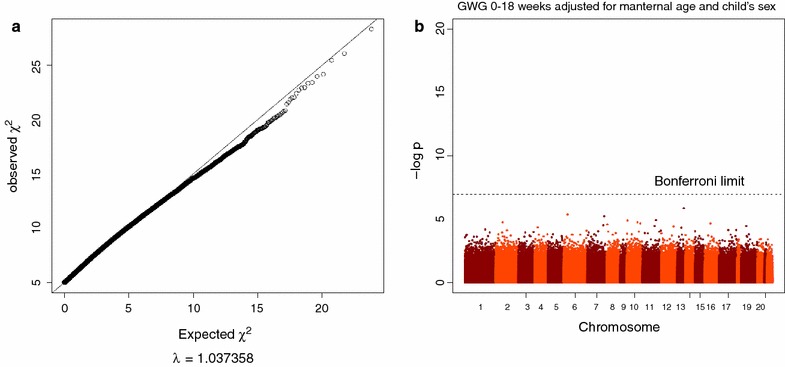
GWG, between weeks 0 and 18. **a** Shows a qq-plot and the genomic inflation factor (λ) for the p values of 0–18 weeks GWG. The p values were taken from a model that was adjusted for child’s sex and maternal age. **b** Is the corresponding Manhattan-plot.

From Fig. 1 it can be seen that the GIF for our 0–18 week GWG model was low: λ = 1.037, but revealed no significant methylation differences after q value correction (*q* > 0.9, 473,731 *tests*, see Table 2; Additional file 2 for more details) [9]. Morales et al. did however run their models on the Illumina GoldenGate Genotyping Assay which maps 1,505 CpG sites, implying that the number of statistical tests corrected for is considerably smaller than the 473,731 tests resulting from our models. Nevertheless, it can be seen from the p values in Table 2 that correcting for multiple testing in accordance with the Illumina GoldenGate Genotyping Assay (1,505 tests) has no consequence for the CpG’s associated with the 0–18 week GWG outcome in our dataset (*q* > *0.9*). Hence, our results indicate that none of the probes were statistically significant for the 0–18 week GWG model.

**Table 2:** Results for 0–18 weeks GWG adjusted for maternal age and child’s sex

Gene (# CpG’s)	GoldenGate CpG	Closest CpG	Coef	Se	GWG p value (adjusted)	GWG q value (450K)	GWG q value (GG)
MMP7* (7)	cg10521988	cg04059146	0.00706	0.00466	0.1300039 (0.9100273)	0.9198694	0.9244006
MMP7 (7)	cg20645973	cg20645973	−0.00141	0.005456	0.7955276 (~1.0)	0.9600729	0.9720421
KCNK4 (26)	cg01352108	cg01352108	−0.00794	0.004879	0.1041649 (~1.0)	0.9198694	0.9244006
KCNK4 (26)	cg25881850	cg25881850	−0.00386	0.004814	0.4233765 (~1.0)	0.9373223	0.9244006
TRPM5 (46)	cg01581060	cg01581060	−0.00152	0.004361	0.7282958 (~1.0)	0.9568195	0.9720421
TRPM5* (46)	cg03982381	cg04691795	−0.01572	0.00626	0.0122687 (0.5643602)	0.9198694	0.9244006
TRPM5* (46)	cg19265431	cg10172068	−0.02234	0.012938	0.012938 (0.595148)	0.9198694	0.9244006
NFKB1* (19)	cg06930505	cg00790535	−0.0176	0.007064	0.0129537 (0.2461203)	0.9198694	0.9244006
NFKB1* (19)	cg24413435	cg12615753	0.00936	0.006383	0.1430112 (~1)	0.9198694	0.9244006

The table shows coefficient estimates (Coef), standard errors (Se) and p/q value estimations for the CpG loci linked to the genes found significantly differentially methylated in the ALSPAC study. The p values are “raw” in the sense that they are unadjusted for multiple testing; p values Bonferroni-adjusted for multiple testing according to the number of CpG’s on the Illumina Humanmethylation450 platform associated with each respective gene is included in brackets. The q values were based on multiple test corrections performed for both the HumanMethylation450 platform [GWG q value (450K), 473,731 tests] and the Golden Gate platform [GWG q value (GG) 1,505 tests]. CpGs marked with * were not directly observed but flanking markers are reported which were associated within the gene body (all *-marked CpG’s linked to the NFKB1 and TRPM5 genes) and promoter regions (*-marked CpG linked to the MMP7 gene).

There were substantially more CpG loci associated with each gene: *MMP7*, *KCNK4*, *TRPM5* and *NFKB1* on the Illumina HumanMethylation450 BeadChip platform than on the Illumina GoldenGate Assay used by Morales et al. [5], but 5 were missing (Table 2). The five CpGs missing in our study were typically replaced by several CpG loci nearby (see Table 2; Additional file 2). The HumanMethylation450 platform contained a total of 98 CpGs, most of which were located in the promoter region of the genes: *MMP7*, *KCNK4*, *TRPM5* and *NFKB1,* and a strong association has been established between the HumanMethylation450 BeadChip and the GoldenGate Assay, of which the former is a more recent evolution rooted in the latter’s technology [10]. It has also been argued that regions spanning several CpGs tend to be differentially methylated as opposed to individual nucleotides therefore we would anticipate several proximate CpG’s to be associated with specific genes [11].

### Quantifying biases that affect the methylome

One factor known to influence DNA methylation is gender, and this has also been observed in the autosomes [12]. Including child’s sex as the only covariate in our 0–18 weeks GWG model did not result in any notable differences, although the GIF decreased from λ = 1.074 for the crude model without covariates, to λ = 1.059. Several studies have indicated that cell types, especially those found in cord blood, may substantially influence the methylome as well [13, 14]. In our study we found that controlling for cell type proportions, using a method described by Houseman et al. [14], improved the model marginally with respect to GIF (λ = 0.998) compared with a crude model (λ = 1.074). However, including cell-type proportions did not lead to any differences with respect to significant CpG loci in any of the models tested, including the full model discussed above of which the GIF increased from λ = 1.042 to λ = 1.206. Another issue that may bias or influence results is the quality-control performed on the methylome dataset. It has been shown that different filtering procedures could influence p values if the signal is weak in potential findings [15].

### Strengths and weaknesses of the present study

Our study strengths include a larger number of newborns than the ALSPAC study and genome-wide coverage of CpG loci. It can however not be concluded that there are no effects of GWG on the methylome since the total methylome in the human genome is assumed to consist of approximately 28 million loci [16], as compared to the 485,512 loci mapped by the HumanMethylation450 platform. Moreover, the mean weight gain approximately 18 weeks after conception (kg/week) for the mothers in our study (0.16 ± 0.17, mean ± SD) was considerably less than for the mothers in the ALSPAC study (0.32 ± 0.17, mean ± SD). Statistical power was also limited for small effect sizes; based on a post hoc power calculation assuming a regression model with 0–18 week GWG, child’s sex, maternal age and cell-type correction (9 explanatory variables in total) we achieved a power of 60% for median effect sizes (*R*^2^ = 0.0610). Small effects sizes (lower quartile, *R*^2^ = 0.0124) resulted in a power below 1% (*R*^2^ refers to the proportion of variance of the corresponding methylation site explained by the variance of the regression model).

## Methods

### Study population

The Norwegian Mother and Child Cohort Study (MoBa) is a prospective population-based pregnancy cohort study conducted by the Norwegian Institute of Public Health [17, 18]. MoBa recruited pregnant women between 1999 and 2008, at approximately 18 weeks after conception and mothers could participate with more than one pregnancy, resulting in 95,200 mothers and 114,500 children in total. The participation rate of invited pregnant women was 40.6%. Umbilical cord blood samples were collected at delivery and sent by post to the biobank at the Norwegian Institute of Public Health where DNA was extracted and stored at −20°C until analysis [19]. The dataset used in the present study is a subset of the one described by Joubert et al. [1]. From that dataset a sub-sample was extracted consisting of a random sample of children (729 in total), included in the analyses by Joubert et al., but children sampled because they had asthma at 3 years were excluded.

The MoBa cohort was linked to the Medical Birth Registry of Norway for information on sex of the child and maternal age. We used information from questionnaires completed by MoBa participants around week 18 of their pregnancies to calculate maternal pre-pregnancy weight (kg), and GWG (kg/week) based on the difference between the mother’s self-reported pre-pregnancy weight and current weight at 18 gestational weeks.

The MoBa study has been approved by the Regional Committee for Medical and Health Research Ethics, the Norwegian Data Inspectorate and the Institution Review Board of the National Institute of Environment Health Sciences, USA. Written informed consent was provided by all participants.

### DNA methylation technology

Cord blood DNA methylation was measured using the Illumina Infinium HumanMethylation450 BeadChip (450K) (http://www.illumina.com). This assay was designed to conduct epigenome-wide association studies (EWAS), and includes 485,577 methylation (CpG) loci per sample at single-nucleotide resolution. This chip covers 99% of RefSeq genes, an average of 17 CpG sites per gene region across the promoter, 5′UTR, first exon, gene body, and 3′UTR. Further, the chip covers 96% of CpG islands, with additional coverage of island shores. Details regarding quality control can be found in the study by Joubert et al. [1] and is also outlined in Additional file 3.

### Statistical analysis

The statistical analyses were performed using standard ordinary least squares regression on each autosomal CpG locus, effectually resulting in 473,731 regression models with 0–18 weeks GWG as the explanatory variable.

The genomic inflation factor (GIF) λ [6] for these models was calculated using the “regression”-based method (default) in the GenABEL package [20].

We used Bonferroni correction and q values to correct for multiple testing [9]. Covariates included in the adjusted model were child’s sex and maternal age (continuous). In addition, we estimated cell type proportions (i.e. CD4, CD8, Gran, NK, Bcell, Mono) using a method suggested by Houseman et al. [14] as implemented in the minfi package [21]. These estimates were then added to the regression model described above as separate covariates. Several other covariates were also tested, and Additional file 1 contains qq-/Manhattan plots of the p values from a model containing the covariates: child’s sex, maternal age, maternal education, maternal daily smoking during pregnancy, caesarian section and parity, (see Table 1 for summary information regarding these covariates) however no differences were detected and therefore the simple model proposed by Morales et al. [5] was presented as the main model.

## Additional files

Additional file 1: The file contains a regression model diagnostics plot including a qq-plot, distribution plot and a manhattan plot of p-values resulting from a regression model based on 0-18 week GWG with covariates child’s sex, maternal daily smoking, maternal education, caesarian section, parity and maternal age. Additional file 2: A table in Microsoft Excel format containing information regarding the regression model based on 0-18 week GWG. Additional file 3: Details regarding quality control of the methylation dataset.
