# Supplementary figures and images for: Effect of maternal gestational weight gain on offspring DNA methylation: a follow-up to the ALSPAC cohort study
Source: BMC Res Notes. 2015 Jul 29;8:321. doi: 10.1186/s13104-015-1286-6 (PMC4518864; doi:10.1186/s13104-015-1286-6)

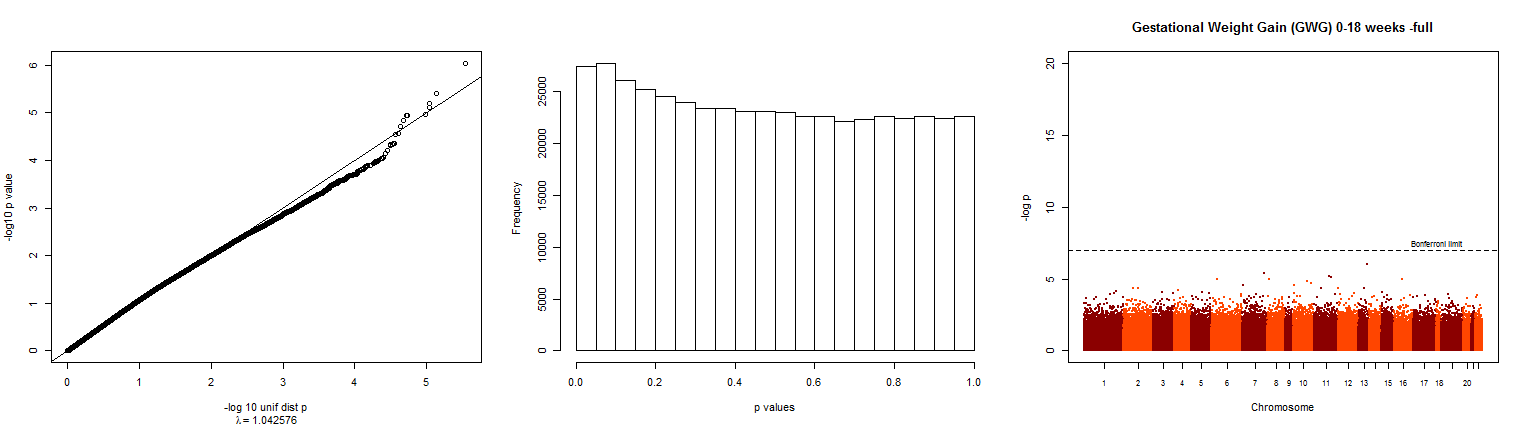

Supplement: Additional file 1: — The file contains a regression model diagnostics plot including a qq-plot, distribution plot and a manhattan plot of p-values resulting from a regression model based on 0-18 week GWG with covariates child’s sex, maternal daily smoking, maternal education, caesarian section, parity and maternal age. [file 13104_2015_1286_MOESM1_ESM.png]
