# Supplementary material for: Effect of maternal gestational weight gain on offspring DNA methylation: a follow-up to the ALSPAC cohort study
Source: BMC Res Notes. 2015 Jul 29;8:321. doi: 10.1186/s13104-015-1286-6 (PMC4518864; doi:10.1186/s13104-015-1286-6)
Supplement: Additional file 3: — Details regarding quality control of the methylation dataset. [file 13104_2015_1286_MOESM3_ESM.docx]

# Details regarding quality control of the Illumina HumanMethylation450 Beadchip based dataset

The original dataset consisted of 1204 samples and 485577 CpG probes. All duplicates (including 13 technical replicates) were removed and only singular children were allowed. The EZ-96 DNA Methylation Kit was used to carry out bisulfite conversion, according to manufacturer recommendations. After removing the 65 Illumina control probes, arrays with more than 5% probes missing, as well as the gender chromosomes and all probes of low detection p value (p value threshold set at 10^-5^) the total number of samples was 1068 with 473731 corresponding CpG probes. All SNP related CpG’s were included in the dataset. BMIQ within-array normalization was performed to calibrate the differing signals of the type I and type II probes [[1](#_ENREF_1)]. In addition, ComBat normalization [[2](#_ENREF_2)] was applied to the dataset to reduce plate effects. Since the 1068 samples contained a larger than expected fraction of asthma cases, a subset consisting of a random sample of 729 children was used in the analysis. More details regarding quality control can be found in the article that first used the methylation dataset used here [[3](#_ENREF_3), [4](#_ENREF_4)].

1. Teschendorff AE, Marabita F, Lechner M, Bartlett T, Tegner J, Gomez-Cabrero D, Beck S: **A beta-mixture quantile normalization method for correcting probe design bias in Illumina Infinium 450 k DNA methylation data**. *Bioinformatics* 2013, **29**(2):189-196.

2. Leek JT, Johnson WE, Parker HS, Jaffe AE, Storey JD: **The sva package for removing batch effects and other unwanted variation in high-throughput experiments**. *Bioinformatics* 2012, **28**(6):882-883.

3. Joubert BR, Haberg SE, Nilsen RM, Wang X, Vollset SE, Murphy SK, Huang Z, Hoyo C, Midttun O, Cupul-Uicab LA *et al*: **450K epigenome-wide scan identifies differential DNA methylation in newborns related to maternal smoking during pregnancy**. *Environmental health perspectives* 2012, **120**(10):1425-1431.

4. Wu MC, Joubert BR, Kuan PF, Haberg SE, Nystad W, Peddada SD, London SJ: **A systematic assessment of normalization approaches for the Infinium 450K methylation platform**. *Epigenetics : official journal of the DNA Methylation Society* 2014, **9**(2):318-329.
